# Decoding Interfacial Polymerization Through Steady Amine Delivery and Real‐Time QCM‐D Monitoring

**DOI:** 10.1002/advs.78089

**Published:** 2026-09-29

**Authors:** Yu Fang, Yu‐Ren Xue, Cheng‐Ye Zhu, Hong‐Qing Liang, Zhi‐Kang Xu

**Affiliations:** ^1^ MOE Key Lab of Macromolecular Synthesis and Functionalization, and Zhejiang Key Laboratory of Advanced Organic Materials and Technologies Department of Polymer Science and Engineering Zhejiang University Hangzhou China; ^2^ The “Belt and Road” Sino‐Portugal Joint Lab on Advanced Materials International Research Center For X Polymers Zhejiang University Haining China

**Keywords:** interfacial polymerization, interfacial stoichiometry, polyamide membranes formation, polymerization kinetics, quartz crystal microbalance with dissipation

## Abstract

Interfacial polymerization (IP) is central to the fabrication of high‐performance polyamide membranes, yet the microscopic reaction pathways governing film formation remain difficult to resolve due to the strongly coupled diffusion‐reaction behavior at the organic‐aqueous interfaces. Here, we develop a tailored IP platform in which a solid‐state piperazine source delivers amine monomers at a constant dissolution flux, thereby decoupling monomer diffusion from polymerization. Combined with in situ quartz crystal microbalance with dissipation monitoring, this system enables real‑time tracking of polyamide formation on the organic side of the reaction front. The frequency‐dissipation response resolves three kinetic regimes: pre‐gelation oligomerization, post‐gelation aggregation, and diffusion‐limited growth accompanied by late‐stage densification. By systematically tuning the interfacial functional‐group ratio, we reveal that the initial stoichiometry governs gelation time, nascent‐network permeability, and subsequent film stiffening, which collectively determine the structure of the final polyamide selective film. Consequently, controlling interfacial reaction conditions enables systematic tuning of the membrane molecular weight cutoff, surface charge, and ion selectivity. This work provides a kinetic map of interfacial polymerization that connects monomer stoichiometry, film‐formation pathways, and membrane structure, offering a mechanistic basis for rationally controlling polyamide membrane formation.

## Graphical abstract

We combine steady amine delivery with in situ quartz crystal microbalance with dissipation to track interfacial polymerization in real time. The platform resolves three kinetic regimes: pre‐gelation oligomerization, post‐gelation aggregation and network growth, and diffusion‐limited film evolution. Initial stoichiometry governs gelation, nascent‐network permeability, and late‐stage stiffening, thereby shaping the final membrane structure.

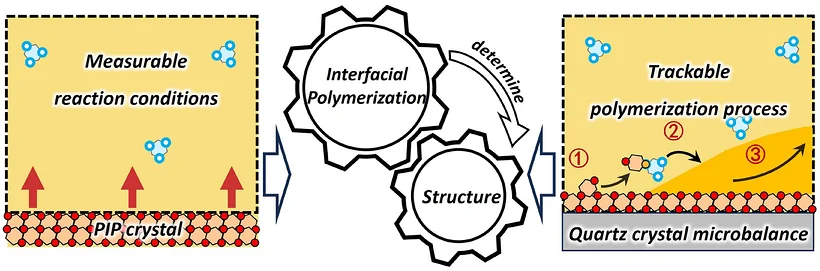

## 1 Introduction

Interfacial polymerization (IP) has become a key manufacturing strategy for ultrathin selective films that underpin modern nanofiltration and reverse‐osmosis membranes [1, 2]. Its distinctive advantage lies in confining polymer formation to the interface between two immiscible phases, allowing highly reactive monomers to generate continuous polymer layers with nanometer‐scale thickness. In a typical IP process, an amine monomer dissolved in water diffuses toward an organic phase containing an acyl chloride monomer and reacts rapidly near the liquid‐liquid interface. The resulting ultrathin polyamide film, supported on a porous substrate, serves as the molecularly selective barrier that governs water and solute transport. This simple yet powerful interfacial process has enabled membrane technologies for seawater desalination, wastewater purification, resource recovery, and other energy‐efficient molecular separations [3, 4, 5]. The separation performance of these membranes is governed by how the polyamide network forms and evolves during IP. Its thickness, cross‐linking, free volume, surface chemistry, and nanoscale heterogeneity collectively determine membrane permeability, selectivity, and long‐term process viability, thereby shaping the performance and economics of large‐scale desalination and wastewater‐treatment technologies [6, 7, 8, 9, 10]. Despite more than half a century of development and broad industrial implementation, IP remains mechanistically opaque [11, 12, 13, 14]. Membrane optimization is still largely guided by recipe‐dependent structure‐performance relationships, while the local diffusion‐reaction pathways that govern nanoscale film formation remain insufficiently quantified [15, 16, 17].

This mechanistic uncertainty stems from the fact that IP occurs within a buried, nanoscale reaction zone where monomer diffusion, chemical reaction, oligomer aggregation, and phase separation proceed simultaneously [18, 19, 20]. The widely accepted model proposed by Freger et al. describes IP as a three‐stage process involving (i) monomer diffusion and reaction, (ii) oligomer nucleation and gelation, and (iii) diffusion‐limited network growth accompanied by phase separation [21, 22]. This model provides a valuable conceptual framework for understanding polyamide film formation, but direct experimental validation has been severely limited. The primary challenge lies in the inability to precisely define the initial reaction conditions at the interface and to decouple the intertwined processes of monomer supply, chemical reaction, and film formation in real time. Consequently, key kinetic parameters, such as the true interfacial monomer concentration, the onset of gelation, and the rates of different reaction stages, have remained inaccessible to direct measurement [23, 24].

Several in situ techniques have been developed to probe IP, including pendant drop tensiometry [25], light reflectometry [26], microfluidics [27, 28], and in situ spectroscopy [29, 30, 31, 32]. These approaches have provided valuable macroscopic information on film growth tendencies, interfacial tension changes, and bulk‐solution monomer depletion. However, their indirect nature fundamentally limits the ability to interrogate the earliest, most critical events occurring at the nanometer‐thick reaction front. Most existing measurements are performed tens to hundreds of micrometers away from the reaction interface, leaving it unclear how local monomer flux, interfacial stoichiometry, and nascent network structure collectively dictate the evolution from dissolved oligomers to cross‐linked polyamide. As a result, the relationship between the prescribed bulk monomer concentrations and the actual local stoichiometry at the reaction front remains poorly defined, limiting rational control over polymer structure and properties [33].

A quantitative understanding of IP therefore requires an experimental platform capable of (i) precisely defining the initial monomer supply, (ii) decoupling monomer diffusion from chemical reaction, and (iii) directly monitoring polymer formation within the interfacial organic phase. Conventional liquid‐liquid IP is poorly suited to this task because amine transport from the aqueous reservoir continuously changes as the reaction proceeds. Under such conditions, changes in initial stoichiometry, diffusion, and network formation are difficult to isolate, and the early events that determine the final film structure are largely obscured.

In this work, we establish a controlled interfacial polymerization platform that enables the initial monomer supply and the subsequent polymerization process to be independently regulated and directly monitored. By replacing the conventional aqueous amine reservoir with a solid piperazine (PIP) source, a constant amine dissolution flux at the initial stage is achieved, allowing the interfacial amine concentration and functional‐group stoichiometry to be quantitatively defined. Coupling this platform with in situ quartz crystal microbalance with dissipation (QCM‐D) monitoring enables real‐time tracking of the complete polyamide formation process (Figure 1 and Figure S1). Two key kinetic transition points are identified, namely the gelation time and the limiting transition time, which divide the process into pre‐gelation oligomerization, post‐gelation aggregation, and diffusion‐limited growth. Systematic variation of the interfacial monomer ratio reveals that the early‐stage stoichiometry governs the nascent network formation and the amine transport, whereas the acyl chloride‐rich conditions trigger a previously overlooked late‐stage densification process. These stoichiometry‐dependent kinetic pathways are consistent with the observed variations in the final structure of the polyamide perm‐selective film, enabling the molecular weight cut‐off, surface charge, and ion selectivity of nanofiltration membranes to be rationally tuned. More importantly, by decoupling a defined monomer‐supply boundary condition from the evolving polymerization process and simultaneously monitoring film formation in real time, this platform transforms IP from an empirically optimized process into an experimentally testable kinetic system. It defines measurable kinetic landmarks for elusive network formation stages and elucidates how early interfacial reaction conditions propagate through monomer transport and structural evolution to dictate ultimate membrane performance. This work provides a robust mechanistic foundation for pathway‐guided polyamide membrane design and offers general applicability to the controllable fabrication of functional polymer films via confined interfacial rapid reactions.

**FIGURE 1:**
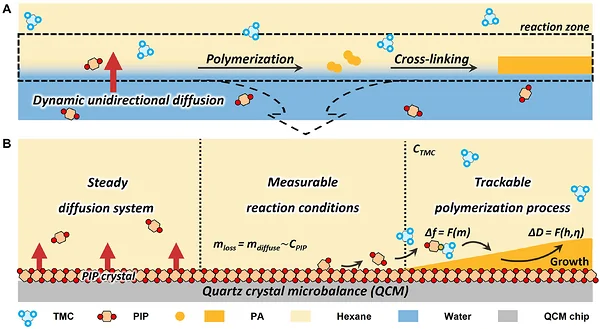
Establishment of an idealized experimental model for interfacial polymerization. (A) Schematic diagram of the interfacial polymerization process at a conventional water‐alkane interface where amine diffusion and polymerization are dynamically coupled. (B) Schematic diagram of the interfacial polymerization process at a solid amine‐alkane interface. The solid amine‐alkane interface provides a constant piperazine (PIP) supply while preserving polymerization in the organic phase, enabling direct monitoring of monomer delivery, network formation, and film growth by quartz crystal microbalance with dissipation (QCM‐D).

## 2 Results and Discussion

### 2.1 Construction of a Controlled Interfacial Polymerization Monitoring System

A solution‐process method using highly volatile hexane was employed to fabricate the stable and uniform PIP layers. Upon solvent evaporation, PIP nucleated and grew at the hexane‐air interface [34], and was subsequently deposited onto the substrate to form a continuous solid layer (Figure S2). Because PIP is prone to sublimation and hydration, all fabrication steps were performed under a controlled atmosphere (Figure S3). Systematic optimization revealed that a temperature of 25°C and relative humidity of 20% provided suitable conditions for the consistent precipitation of PIP (Figure S4 and Table S1). Under these conditions, the formed PIP crystals exhibited a distinct unimodal distribution of grain sizes (Figure S5), facilitating the formation of a smooth and uniform amine layer. The gravimetric analysis showed that the load of the solid amine layer on the substrate surface gradually increased with the volatilization time (Figure S6). Even after only 0.5 min of deposition, the PIP loading exceeded 100 ng cm^−2^, far beyond the theoretical amount required for the IP reaction, indicating that the solid amine layer can provide a sufficient monomer reservoir. White light interferometry further demonstrated that the surface roughness of the solid amine layer approached a stable value with increasing evaporation time, confirming the reproducibility of the fabricated layers (Figure S7).

The key advantage of this methodology is that the initial amine supply can be defined quantitatively. Since PIP enters the organic phase only through dissolution of the solid layer at the interface, the participating amine flux can be determined from the mass loss of the solid PIP (Figure S8). In situ QCM measurements of PIP dissolution in hexane confirmed that the dissolution rate remained constant during the first 3 s after contact with hexane (Figure 2A; Figures S9 and S10). This timeframe is significantly longer than the characteristic timescale of the early IP reaction [15]. Since the consumption of reactive monomers at the reaction front is expected to be faster than their supply under diffusion‐controlled conditions, PIP delivery in our system can be approximated as a constant‐flux process in the early stages of the reaction. COMSOL multiphysics simulations further indicated that this constant dissolution flux corresponds to a steady‐state interfacial amine monomer concentration of 11.6 mmol L^−1^ at the interface (Figure S11).

**FIGURE 2:**
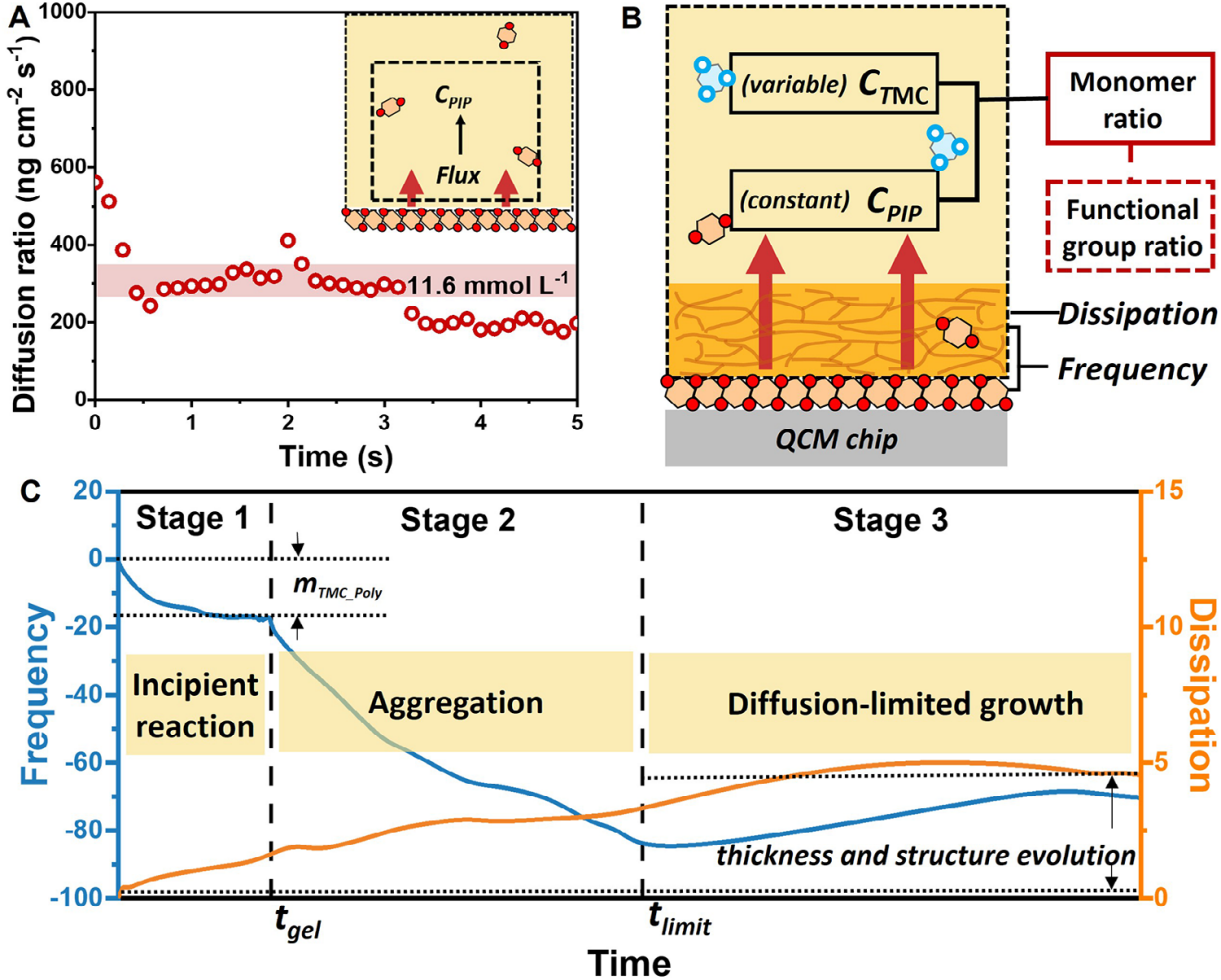
(A) Constant‐rate diffusion behavior of solid piperazine (PIP) in hexane, (B) Control of monomer ratio at the interface by varying trimesoyl chloride (TMC) concentration, and (C) In situ quartz crystal microbalance with dissipation monitoring of the entire IP process with assignment of the gelation time (*t*_gel_), limiting transition time (*t*_limit_), and three kinetic stages.

With the interfacial amine concentration fixed, the initial reaction stoichiometry can be manipulated by varying only the trimesoyl chloride (TMC) concentration in hexane (Figure 2B). Because PIP is difunctional and TMC is trifunctional, the monomer ratio is further expressed as a chemically more meaningful amino/acyl chloride functional‐group ratio. The ratios examined here span amino‐terminated (2:1), fully cross‐linked (1:1), linear (1:1.5), and acyl chloride‐terminated structures (1:3). This design enables an in‐depth and systematic investigation of how interfacial stoichiometry determines the kinetic pathway and final polyamide structure (Figure S12). It should be noted that these functional‐group ratios represent defined reaction conditions in the present model system rather than the nominal bulk formulations used in conventional liquid‐liquid IP. In practical IP, even when PIP is present in excess in the aqueous phase, progressive restriction of amine transport through the nascent polyamide layer may shift the local reaction environment toward relative acyl chloride excess. The acyl chloride‐rich conditions examined here therefore serve to probe such limiting local stoichiometric states rather than to reproduce specific commercial formulations.

Figure 2C provides the central kinetic map of the IP process from in situ QCM‐D monitoring. As shown in the figure, changes in the slope of *Δf* define two transition points, dividing the process into three distinct stages. Stage I is assigned to pre‐gelation oligomerization. Immediately after TMC/hexane contacts the solid PIP layer, PIP dissolves into the organic phase at a constant flux and reacts with TMC near the sensor surface to generate soluble or weakly associated oligomers. During this stage, *f* decreases rapidly because the growing oligomer/polyamide species increase the coupled mass on the sensor, whereas *D* increases because the deposited layer is soft and highly viscoelastic. The first transition point is defined as the gelation time (*t*_gel_), at which these oligomers connect into a percolated polyamide network.

Stage II corresponds to the post‐gelation aggregation. Between *t*_gel_ and the second transition point, the gelled network continues to capture monomers and oligomers, followed by local aggregation/phase separation, resulting in a rapid increase in its viscoelastic contribution. This process produces a second rapid decrease in *f*, together with a continued or accelerated increase in *D*, reflecting simultaneous mass incorporation and viscoelastic network formation. The second transition point is defined as the limiting transition time (*t*_limit_), marking the moment when the continuous polyamide layer becomes the dominant barrier to further PIP transport, and the system enters diffusion‐limited growth.

Stage III is assigned to diffusion‐limited growth. After *t*_limit_, additional PIP transport through the nascent polyamide layer becomes increasingly restricted. As a result, mass uptake slows down, *D* gradually approaches a plateau, and *f* can partially recover or increase. This late‐stage *f* recovery and *D* decrease are assigned to network stiffening and densification rather than simple mass loss [22]. Overall, the QCM‐D response directly resolves the transition from oligomer formation to gelation, reaction‐limited network growth, and diffusion‐limited film evolution. It aligns with and provides experimental validation for Freger's three‐stage theoretical model of IP film formation, offering direct insight into the kinetic transitions of the reaction.

### 2.2 Stoichiometric Regulation of Early Gelation and Nascent‐Layer Transport

With the kinetic stages assigned in Figures 2C and 3 focuses on how interfacial stoichiometry regulates the earliest stage of polyamide formation. Stage I determines the initial network topology before the film becomes a significant diffusion barrier. Therefore, changes during this stage are expected to propagate into later mass transport, film growth, and final membrane properties.

**FIGURE 3:**
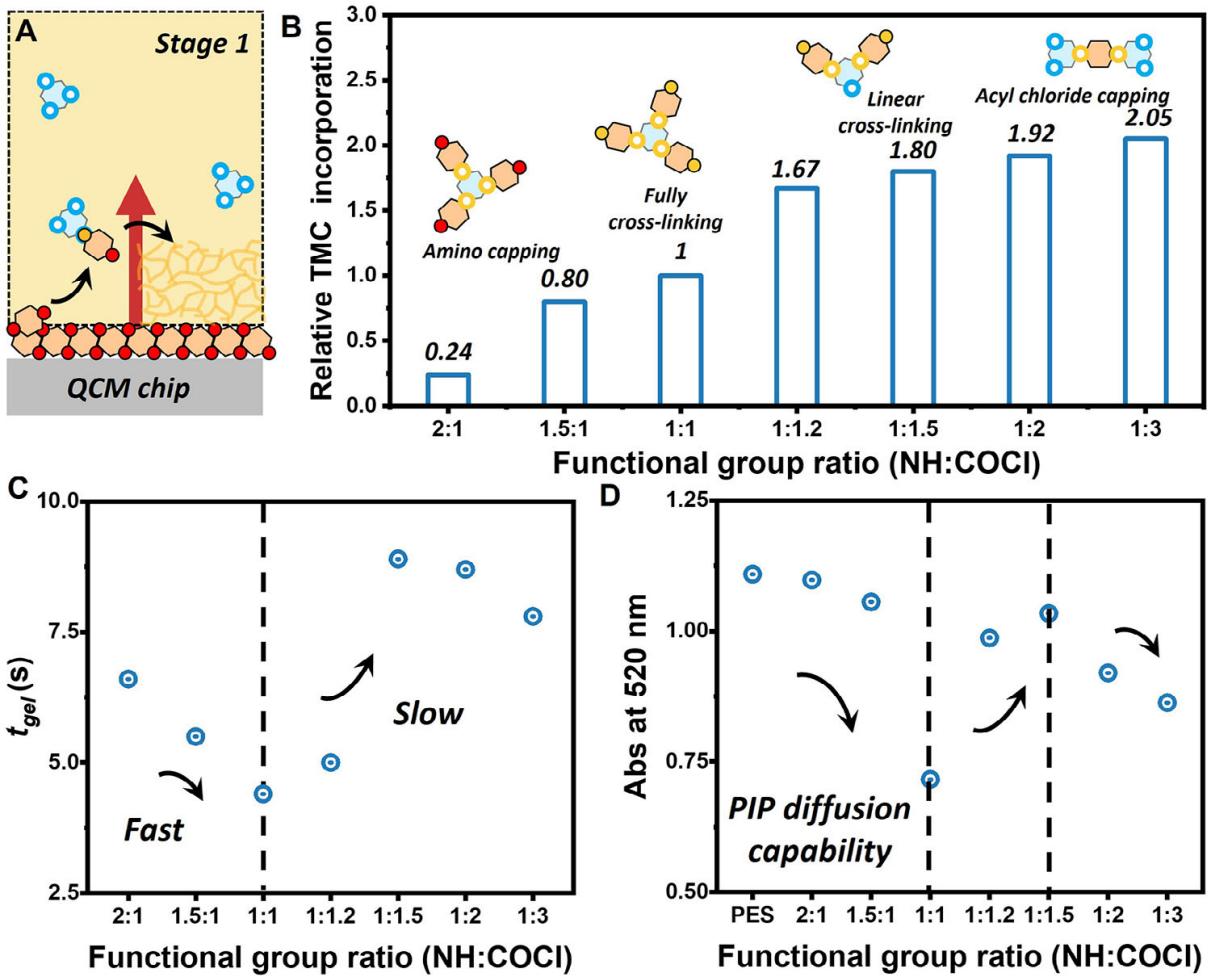
Stoichiometric regulation of Stage I polymerization. (A) Schematic illustration of early‐stage oligomerization and gelation. (B) Relative trimesoyl chloride (TMC) incorporation as a function of the functional‐group ratio estimated from the frequency response. Herein, the relative TMC incorporation is calculated as the ratio of the consumed TMC mass at different functional‐group ratios to that at the equimolar ratio. (C) Gelation time as a function of the functional‐group ratio, and (D) UV absorbance of piperazine (PIP) after diffusion across the nascent layer during Stage I.

During Stage I, the corresponding decrease in *f* signals the incorporation of TMC‐containing species into the developing polyamide layer, while the increase in dissipation (Δ*D*) indicates that the newly formed layer is viscoelastic rather than rigid (Figures 3A and S13). At this nascent phase, the system can be approximated by a simplified model. Here, three assumptions were made: (i) all dissolved PIP participates in the polymerization reaction. (ii) the dissolution behavior and mechanical properties of early‐stage polymers are comparable across different stoichiometries. (iii) the dissipation changes are neglected during the initial stage. Cause the solid PIP behaves as a rigid mass source, and the QCM‐D response is dominated by the forming polyamide.

Under these assumptions, the frequency shift during Stage I was used to approximate the apparent relative TMC incorporation. To evaluate the impact of reaction stoichiometry on interfacial polymerization reactions, the apparent relative TMC incorporation was calculated under seven reaction functional group ratios (Figure S14). We note that this assumption can lead to an underestimate, particularly under amine‐rich conditions, where a fraction of dissolved PIP may diffuse into the bulk organic phase without reacting. The calculated values should therefore be interpreted semi‐quantitatively as an apparent stoichiometric trend rather than as absolute TMC incorporation masses. Nevertheless, the overall variation provides complementary information on the dependence of the early‐stage reaction on the prescribed functional‐group ratio. Taking the 1:1 functional‐group ratio as a reference, the calculated relative TMC incorporation broadly follows the prescribed initial functional‐group ratio, confirming that the solid‐state amine supply enables meaningful regulation of interfacial stoichiometry (Figure 3B). Correspondingly, *t_gel_* reaches a minimum near the 1:1 ratio (Figure 3C), indicating that balanced amino and acyl chloride groups promote the fastest formation of a percolated polyamide network. Deviation from stoichiometry delays gelation because one functional group becomes locally insufficient for rapid network formation. These results show that the early‐stage QCM‐D response is highly sensitive to the prescribed interfacial stoichiometry.

This stoichiometry‐dependent early network directly governs subsequent amine transport. Measurement of PIP transmembrane diffusion across 10 s‐aged nascent layers reveals a non‐monotonic dependence on functional‐group ratio (Figure 3D). Near the stoichiometric ratio, the nascent network is densest, resulting in the lowest PIP permeability and the smallest early‐stage thickness of 9.3 nm (Figures S15 and S16). Under amine‐rich conditions, insufficient acyl chloride cross‐linking yields a looser nascent layer, thereby facilitating PIP transport. Under acyl chloride‐rich conditions, the network is also initially imbalanced, but the increased layer thickness (17.7 nm) prolongs the transport pathway. In addition, residual acyl chloride groups within the nascent layer provide additional reaction sites for the diffusion of PIP, further suppressing its permeation at the highest TMC ratios. These results highlight that Stage I stoichiometry determines not only the rate of gelation but also the permeability of the nascent film, which subsequently controls later‐stage growth.

### 2.3 Stoichiometric Regulation of Diffusion‐Limited Growth and Film Densification

After gelation, the polymerization enters Stage II and Stage III, during which monomer diffusion becomes increasingly restricted, and oligomers undergo further cross‐linking and precipitation via phase separation. Stage II is short and involves strongly coupled reaction, aggregation, and phase separation. These processes cause pronounced fluctuations in both *f* and *D*, making precise quantitative comparison difficult. In contrast, after approximately 20 s, all systems have entered Stage III, where the QCM‐D response provides a clearer window into diffusion‐limited growth, network stiffening, and film densification.

In Stage III, the QCM‐D response separates into three stoichiometry‐dependent regimes (Figures 4A and S17). Under amine‐rich conditions, *D* increases rapidly and then reaches a plateau (Figure 4B), indicating continued formation of a relatively soft and thick polyamide layer before growth becomes self‐limited by increasing mass‐transfer resistance. Near the stoichiometric ratio, *D* increases more slowly and reaches a lower plateau (Figure 4C). This behavior is consistent with the dense nascent layer formed in Stage I, which suppresses PIP permeation and limits further film thickening. Under acyl chloride‐rich conditions, *D* remains nearly unchanged and can even decrease at longer reaction times (Figure 4D). This response indicates that additional PIP diffusing through the film is consumed by residual acyl chloride groups within the nascent layer, promoting internal cross‐linking and stiffening rather than outward thickness growth.

**FIGURE 4:**
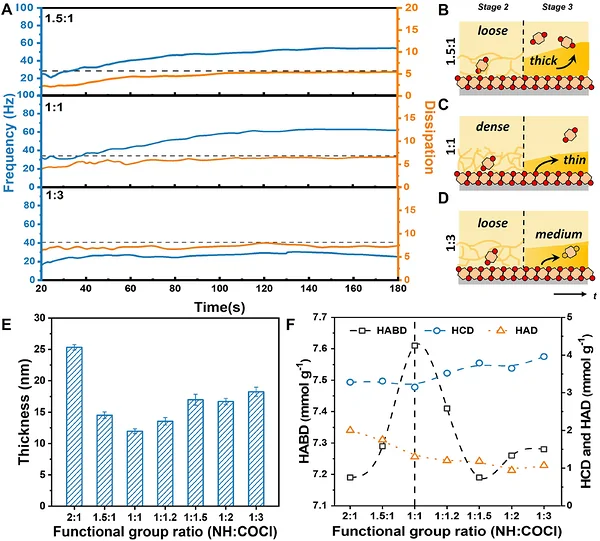
Stoichiometry‐dependent Stage III growth and densification. (A) Frequency and dissipation responses during diffusion‐limited growth. (B–D) Schematic illustrations of late‐stage polymerization pathways under amine‐rich, stoichiometric, and acyl chloride‐rich conditions. (E) Final polyamide film thickness and (F) harmonic amide bond density, harmonic carboxyl density, and harmonic amino density of the fabricated polyamide films.

The thickness measurements support this interpretation (Figure 4E). Amine‐rich films continue to grow during Stage III because the loose nascent network allows sustained PIP transport, ultimately generating thicker polyamide layers. Stoichiometric films remain thin because early gelation produces a compact network that strongly restricts monomer permeation. In the most acyl chloride‐rich case (1:3), the final thickness increases by only about 0.5 nm compared with the nascent layer. This small increase in thickness indicates that late‐stage polymerization is dominated by densification and hardening of the existing network rather than by continuous film thickening.

The chemical composition of the final polyamide films further confirms this stoichiometry‐dependent evolution. XPS‐derived harmonic amide bond density (HABD), harmonic carboxyl density (HCD), and harmonic amino density (HAD) of the membrane prepared at the corresponding functional group ratios are not in simple proportion to the prescribed functional‐group ratio [35] (Figure 4F and Table S2). Under amine‐rich conditions, increasing the proportion of acyl chloride favors more complete reaction of PIP monomers, leading to an increase in HABD and a decrease in HCD and HAD. At the stoichiometric functional group ratio, HABD reaches a maximum of 7.61 mmol g^−1^, and the surface energy reaches its minimum value of 66.44 mN m^−1^, corresponding to the lowest residual surface group under highly cross‐linked conditions (Figure S18). When acyl chloride becomes excessive as the end‐capping condition, HABD first decreases and then increases slightly from 7.19 to 7.28 mmol g^−1^. This recovery of HABD under acyl chloride‐rich conditions confirms the presence of late‐stage internal densification during IP, in which residual acyl chloride groups react with transported PIP within the preformed network.

### 2.4 Kinetic‐Structure‐Performance Correlations

The stoichiometry‐dependent kinetic pathways identified above ultimately translate into tunable membrane properties. The initial functional‐group ratio controls early gelation, nascent‐layer permeability, and late‐stage growth/densification, thereby providing a direct handle for adjusting the microstructure, surface charge, and separation performance of the final polyamide membranes. The membrane‐performance measurements are used here to evaluate the structural consequences of the different formation conditions rather than as independent proof of the proposed kinetic pathway.

The late‐stage densification observed under acyl chloride‐rich conditions has a particularly strong influence on the internal architecture of membranes. This effect is quantitatively reflected in the molecular weight cut‐off (MWCO) of the synthesized membranes. As the TMC ratio increases, the MWCO gradually decreases from 505 to 351 Da (Figure 5A). This trend is notable because the acyl chloride‐rich membranes exhibit tighter size sieving even though their nominal surface cross‐linking density is lower than that of the stoichiometric membranes. The apparent discrepancy can be rationalized by the densification pathway identified in Figure 4: residual acyl chloride groups within the nascent film react with subsequently transported PIP, forming a denser internal network that dominates molecular sieving. Consequently, amine‐rich conditions favor looser and more permeable films, whereas acyl chloride‐rich conditions favor tighter nanofiltration films through late‐stage internal densification.

**FIGURE 5:**
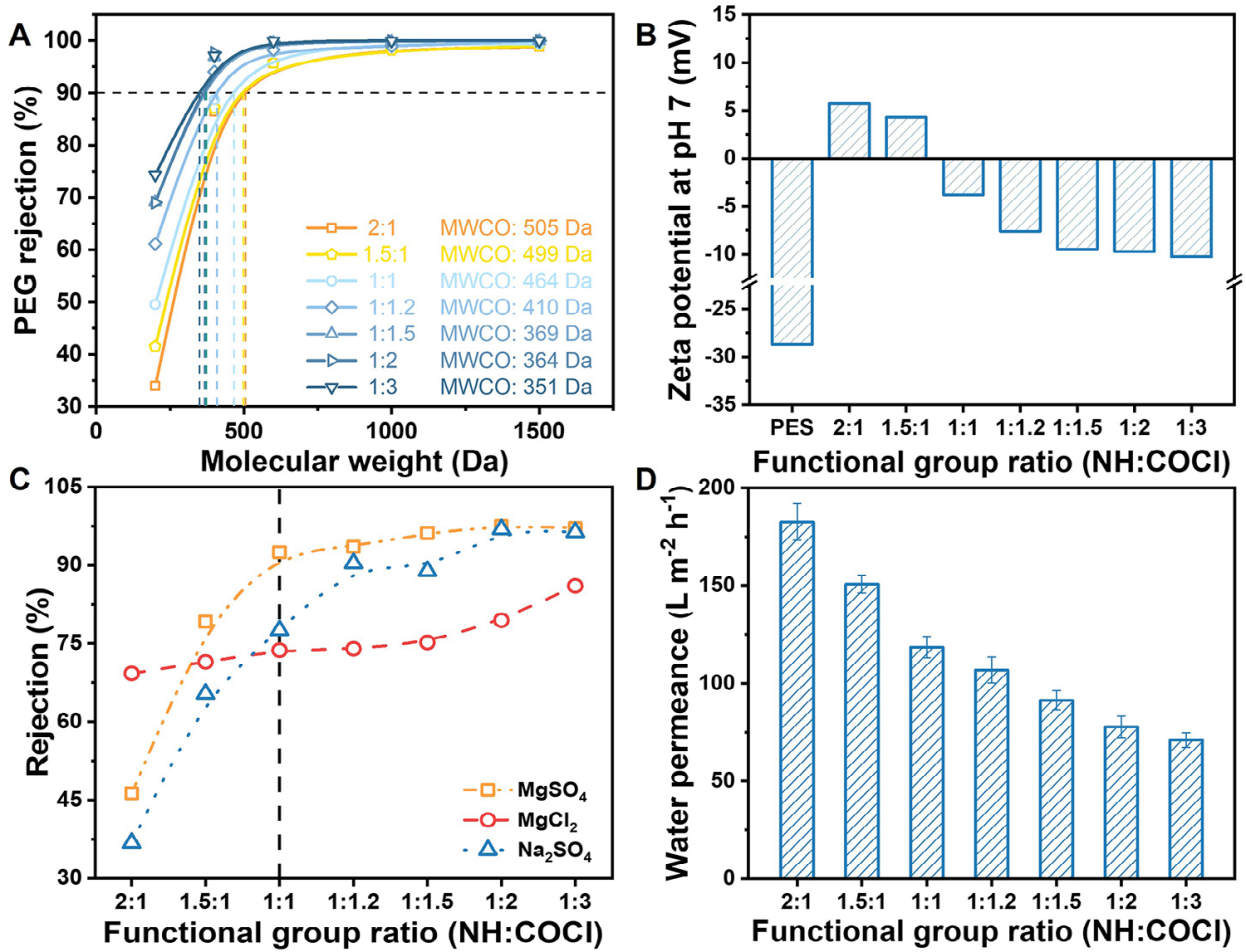
Separation properties of the resulting composite membranes. (A) Molecular weight cutoff (MWCO), (B) Zeta potential, (C) Rejection performance of different salts, and (D) Water permeance of the composite membranes.

Surface morphology and charge are also governed by the initial functional group ratio. SEM images show that the number and size of vesicular surface structures vary with reaction stoichiometry (Figure S19). The vesicular surface structures are likely related to the local accumulation and evolution of HCl generated during amidation. In contrast to conventional liquid–liquid IP, the present solid‐liquid system lacks an aqueous phase capable of dissolving and neutralizing HCl. As a result, HCl accumulates between the solid amine layer and the growing polyamide layer, leading to vesicle formation. The observed vesicles therefore represent a model‐specific consequence of the altered HCl‐removal pathway and should not be interpreted as a direct reproduction of commercial membrane morphology. Importantly, however, this difference does not eliminate the formation of a polyamide transport barrier or the subsequent diffusion of amine through the growing film, which are the kinetic processes primarily interrogated in this study.

The final surface morphology depends strongly on the structure of the nascent polyamide layer. Near the stoichiometric ratio, rapid formation of a dense nascent layer restricts gas release and limits subsequent reactions, resulting in a surface with small and densely distributed vesicles. As the acyl chloride fraction increases, delayed gelation allows more HCl to be generated before the network becomes compact, producing larger vesicles. At the highest acyl chloride excess of 1:3, the looser nascent layer facilitates gas escape, resulting in a relatively smooth and uniform surface. Zeta potential measurements show a systematic shift in surface charge from +5.73 to −10.26 mV as the TMC fraction increases (Figure 5B), confirming that surface chemistry is directly regulated by the prescribed stoichiometry. Notably, the membrane surface remains slightly positive under amine‐rich conditions, even though the upper surface is exposed to the TMC‐rich organic phase. This indicates that a significant population of unreacted amine groups persists within the polymer network.

The combined changes in pore size and surface charge determine ion‐selective nanofiltration performance. The rejection performance of composite membranes prepared at different functional group ratios was evaluated for MgSO_4_, MgCl_2_, and Na_2_SO_4_ (Figure 5C). Under amine‐rich conditions, the positively charged and relatively loose membranes preferentially reject cations such as Mg^2+^. For example, at a functional group ratio of 2:1, although the overall rejection is limited due to larger pores, MgCl_2_ rejection reaches 69.3%, whereas Na_2_SO_4_ rejection remains only 36.8%. Conversely, under acyl chloride‐rich conditions, the negatively charged and densified membranes preferentially reject divalent anions. Moreover, the reduced pore size enhances overall salt rejection, with the highest Na_2_SO_4_ rejection reaching 96.9%. This behavior reflects the combined contribution of size sieving and Donnan exclusion, demonstrating that interfacial stoichiometry can be used to tailor membrane selectivity toward specific ionic species.

The enhanced selectivity and denser structure achieved under TMC‐rich conditions come at the expense of permeability. The increased transmembrane resistance associated with the densified, thicker polyamide films results in a marked decrease in water flux (Figure 5D). This establishes a clear kinetics‐structure‐performance relationship on the basis of traditional liquid‐liquid interfacial polymerization: interfacial stoichiometry controls the kinetic route of film formation, and this route determines the balance between permeability, pore‐size sieving, and Donnan exclusion (Figure S20). Our results quantitatively allow for the strategic navigation of this performance landscape to design membranes tailored for specific separation needs.

## 3 Conclusion

This study has established a flux‐defined interfacial polymerization platform decoupling amine delivery from reaction, and enabling direct QCM‐D interrogation of polyamide film formation. By employing a solid‐state amine source with an initial constant dissolution flux, the interfacial amine concentration can be quantitatively defined, allowing the functional‐group ratio to be tuned through the TMC concentration. The QCM‐D response has resolved three kinetic stages: pre‐gelation oligomerization, post‐gelation aggregation, and diffusion‐limited growth accompanied by late‐stage densification. Two characteristic transition points, *t*_gel_ and *t*_limit_, respectively mark the formation of a percolated polyamide network and the onset of diffusion‐limited growth. Systematic variation of stoichiometry has revealed that early network formation regulates amine permeability, which in turn dictates whether later polymerization proceeds by film thickening or internal densification. Under acyl chloride‐rich conditions, late‐stage densification produces membranes with smaller effective pores and enhanced ion rejection despite a lower nominal surface cross‐linking density. By linking controlled initial stoichiometry, QCM‐D‐resolved kinetic transitions, chemical structure, and separation performance, this work provides a mechanistic framework for understanding IP at its reactive front and establishes a basis for mechanism‐guided design of polyamide composite membranes. Beyond the PIP‑TMC nanofiltration system, the solid‑source/QCM‑D platform developed here is broadly applicable to other rapidly formed interfacial polymer networks, including polyamide, polyester, polyurea, and covalent organic framework membranes, enabling systematic investigation of how reaction conditions, monomer chemistry, solvent environment, and other factors influence the structure and performance of polymer films. By moving the study of interfacial polymerization beyond empirical formulation‐performance correlations toward quantitative kinetic understanding, this platform provides both fundamental insight and practical guidance for precision molecular separations, sustainable water treatment, and energy‐efficient membrane technologies.

## 4 Experimental Section

### 4.1 In Situ Monitoring of the Dissolution Ratio of the Solid Amine Layer in Hexane via QCM‐D

The gold sensor chip was treated according to the standard cleaning procedure recommended by Biolin Scientific [36]. The experiment was conducted under controlled conditions (25°C, 20% RH) using the open module. Initially, the baseline was stabilized for 20 min in the air environment, ensuring that no noise or drift was present. Subsequently, 1 mL saturated PIP/hexane solution was added to the open module and allowed to stand for 2 min for evaporation. The supernatant was then carefully removed, and the surface residual liquid was allowed to evaporate completely, resulting in the formation of the solid amine layer. Then, hexane was rapidly added, and the frequency and dissipation changes were continuously monitored. The collected data were analyzed using QTools software, with a focus on the signals at the third, fifth, and seventh overtones.

### 4.2 In Situ Monitoring of Interfacial Polymerization via QCM‐D

The in situ monitoring of the solid‐liquid interfacial polymerization followed the procedure similar to that described in the previous section. Once the baseline was stable for 20 min with no noise or drift detected, 1.0 mL saturated PIP/hexane solution was applied to the surface of the gold sensor chip to form the solid amine layer. After complete evaporation of the hexane, 1.0 mL of TMC hexane solution at the desired concentration was rapidly added, and the subsequent changes in the frequency (*f*) and dissipation (*D*) signals were recorded. The monitoring period lasted for 120 min. The collected data were analyzed using QTools software, with a focus on the signals at the third overtones.

During interfacial polymerization, the raw QCM‐D traces exhibited intermittent high‐frequency fluctuations associated with transient mechanical disturbances from HCl generation and release. To extract the underlying long‐timescale evolution of the polymer film, abrupt signal excursions beyond the normal local fluctuation envelope were excluded according to a predefined criterion. For the late‐stage response (>20 s), data were additionally sampled at fixed time intervals for visualization of the baseline trend. Kinetic transition points and mechanistic interpretations were determined from the persistent evolution of the baseline response rather than from isolated transient excursions.

### 4.3 QCM‐D Model for Interfacial Polymerization

The solid amine‐alkane interfacial polymerization system consists of three components, arranged from bottom to top as follows: a solid amine layer composed of small‐molecule PIP, a polymer layer formed by the reaction, and an uppermost layer of TMC/hexane solution (Figure S1). Assuming the gold sensor is layer 0, and the solid amine layer is layer 1, with subsequent layers numbered sequentially, the corresponding layer parameters‐ thickness, viscosity, shear modulus, and density are *h_j_*, *η_j_*, *µ_j_*, and *ρ_j_*, where *j* = 0, 1, 2, 3. Substituting into the theoretical model of QCM‐D, the expressions for frequency shift (*Δf*) and dissipation change (*ΔD*) in the liquid environment with two adsorbed layers can be derived as follows [37, 38, 39]:

(1) $$\begin{array}{c} \Delta f\approx -\frac{1}{2\pi \rho_{0}h_{0}}\left[\frac{\eta_{3}}{\delta_{3}}+\sum_{j=1,2}\left(\rho_{j}h_{j}\omega -2{\left(\frac{\eta_{3}}{\delta_{3}}\right)}^{2}\frac{\eta_{j}\omega h_{j}}{\mu_{j}^{2}+\eta_{j}^{2}\omega^{2}}\right)\right] \end{array}$$

(2) $$\Delta D\approx \frac{1}{\pi \rho_{0}h_{0}f}\left[\frac{\eta_{3}}{\delta_{3}}+\sum_{j=1,2}2{\left(\frac{\eta_{3}}{\delta_{3}}\right)}^{2}\frac{\mu_{j}\omega h_{j}}{\mu_{j}^{2}+\eta_{j}^{2}\omega^{2}}\right]$$

where *ω* is the angular frequency of the quartz crystal resonance, and $\delta_{3}=\sqrt{2\eta_{3}/\omega \rho_{3}}$ is the penetration depth of the shear wave in the liquid. As indicated by the formula, the total responses of frequency and dissipation are the sum of contributions from all layers.

For layer 1, the solid amine layer can be treated as a thin and rigid layer (μ_1_ > >ωη_1_) [40]. Accordingly, the formula can be simplified as follows:

(3) $$\Delta f_{1}\approx -\frac{1}{2\pi \rho_{0}h_{0}}\rho_{1}h_{1}\omega =-\frac{1}{2\pi \rho_{0}h_{0}}\frac{\omega m_{1}}{A}$$

(4) $$\Delta D_{1}\approx 0$$

where *A* is the sensor area, and *m* is the mass. It can be observed that the frequency is inversely proportional to the loaded mass, as described by the Sauerbrey equation.

Similarly, the expressions for the contributions of the second layer to the frequency and dissipation can be derived.

(5) $$\Delta f_{2}\approx -\frac{1}{2\pi \rho_{0}h_{0}}\left[\frac{\omega m_{2}}{A}-2{\left(\frac{\eta_{3}}{\delta_{3}}\right)}^{2}\frac{\eta_{2}\omega h_{2}}{\mu_{2}^{2}+\eta_{2}^{2}\omega^{2}}\right]$$

(6) $$\Delta D_{2}\approx \frac{2}{\pi \rho_{0}h_{0}f}{\left(\frac{\eta_{3}}{\delta_{3}}\right)}^{2}\frac{\mu_{2}\omega h_{2}}{\mu_{2}^{2}+\eta_{2}^{2}\omega^{2}}$$

To simplify the model system, it is assumed here that all of the PIP dissolved in the alkane organic phase participates in the interfacial polymerization reaction. The changes in frequency and dissipation at reaction time *t* relative to the initial time *t_0_* are as follows:

(7) $$\begin{array}{ccc} \Delta \;f_{t-t_{0}} & = & \Delta f_{t,1}+\Delta f_{t,2}+\Delta f_{t,3}-\Delta f_{t_{0},1}-\Delta f_{t_{0},3}\\ & \approx & -\frac{1}{2\pi \rho_{0}h_{0}}\left[\omega \frac{m_{t,1}+m_{t,2}-m_{t_{0},1}}{A}-2{\left(\frac{\eta_{3}}{\delta_{3}}\right)}^{2}\frac{\eta_{2}\omega h_{2}}{\mu_{2}^{2}+\eta_{2}^{2}\omega^{2}}\right]\\ & = & -\frac{1}{2\pi \rho_{0}h_{0}}\left[\frac{\omega m_{tmc-poly}}{A}-2{\left(\frac{\eta_{3}}{\delta_{3}}\right)}^{2}\frac{\eta_{2}\omega h_{2}}{\mu_{2}^{2}+\eta_{2}^{2}\omega^{2}}\right] \end{array}$$

(8) $$\Delta D_{t-t_{0}}=\Delta D_{t,2}\approx \frac{2}{\pi \rho_{0}h_{0}f}{\left(\frac{\eta_{3}}{\delta_{3}}\right)}^{2}\frac{\mu_{2}\omega h_{2}}{\mu_{2}^{2}+\eta_{2}^{2}\omega^{2}}$$

where *m*_tmc‐poly_ is the mass of TMC participating in the interfacial polymerization reaction.

It can be observed that as the reaction progresses, the frequency shift consists of two components: the mass of TMC involved in the reaction and a correction term influenced by the thickness and structural parameters of the formed polymer layer. In contrast, the dissipation is solely affected by the thickness and structure of the generated polymer layer.

### 4.4 Preparation of Polyamide Thin‐film Composite Membranes

Under environmental conditions of 25°C temperature and 20% RH, 5 mL of saturated PIP/hexane solution was added onto the surface of a polyethersulfone ultrafiltration membrane with an effective area of 38.5 cm^2^. The solution was allowed to stand for evaporation for 2 min. Then, the supernatant was decanted, and the membrane was left for 1 min to ensure complete evaporation of the surface liquid layer. Subsequently, TMC solutions of different concentrations were applied and allowed to react for 2 min. After the reaction, excess TMC solution was removed, and the membranes underwent post‐treatment at 60°C for 5 min.

## Author Contributions

**Yu Fang**: Idea construction, Investigation, Data Curation, Formal Analysis, Writing – original draft. **Yu‐Ren Xue**: Methodology, Validation, Discussion. **Cheng‐Ye Zhu**: Discussion, Investigation. **Hong‐Qing Liang**: Discussion, Writing – Review & Editing, Visualization. **Zhi‐Kang Xu**: Project Administration, Supervision, Funding Acquisition.

## Funding

This work was supported by the National Natural Science Foundation of China (Grant No. 22135006), the Fundamental Research Funds for the Central Universities (226‐2024‐00074), and the Open Research Project of the Innovation Center of Yangtze River Delta, Zhejiang University.

## Conflicts of Interest

The authors declare no conflicts of interest.

## Supporting information




**Supporting File**: advs78089‐sup‐0001‐SuppMat.docx.

## Data Availability

The data that support the findings of this study are available from the corresponding author upon reasonable request.
