# Supplementary material for: Decoding Interfacial Polymerization Through Steady Amine Delivery and Real‐Time QCM‐D Monitoring
Source: Adv Sci (Weinh). 2026 Sep 29:e78089. Online ahead of print. doi: 10.1002/advs.78089 (PMC13624326; doi:10.1002/advs.78089)
Supplement: Supplementary file 1 [file ADVS-9999-e78089-s001.docx]

**Supporting Information**

Decoding Interfacial Polymerization through Steady Amine Delivery and Real-time QCM-D Monitoring

*Yu Fang, Yu-Ren Xue, Cheng-Ye Zhu, Hong-Qing Liang^*^, Zhi-Kang Xu^*^*

*^a^ MOE Key Lab of Macromolecular Synthesis and Functionalization, and Zhejiang Key Laboratory of Advanced Organic Materials and Technologies, Department of Polymer Science and Engineering, Zhejiang University, Hangzhou 310058, China*

*^b^ The “Belt and Road” Sino-Portugal Joint Lab on Advanced Materials, International Research Center for X Polymers, Zhejiang University, Haining 314400, China*

**Corresponding author, email:* [liang.hongqing@zju.edu.cn; xuzk@zju.edu.cn](mailto:liang.hongqing@zju.edu.cn;%20xuzk@zju.edu.cn)

**Experimental section**

**Materials**

The gold sensor chip was purchased from Biolin Technology Co., Ltd. (Sweden). Ultrafiltration membrane of polyether sulfone (PES), produced by Beijing Seprit Equipment Co., Ltd. (China), served as porous substrates for the preparation of composite membranes. Piperazine (PIP) and polyethylene glycol (PEG) with various molecular weights (M_n_ = 200, 400, 600, 1000, 1500) were obtained from Aladdin Chemical Co., Ltd. (China). Trimesoyl chloride (TMC) was purchased from Qingdao Benzo Chemical Co., Ltd. (China). Additional chemicals, such as hexane, sodium sulfate, magnesium chloride, and magnesium sulfate, were purchased from Sinopharm Chemical Reagent Co., Ltd. (China). All reagents were used without further purification. The ultrapure water (18.2 MΩ) was directly produced by an ELGA water system (France).

**Characterizations**

X-ray photoelectron spectroscopy (Thermoscientific, K-Alpha+, USA) were applied to reveal the surface chemistry of composite membranes. The calculation of HABD, HAD, HCD followed the method established in previous literature^1^. The surface charges of all membranes were measured using the streaming-potential method by an electric analyzer (SurPASS Anton Paar, GmbH, Austria). The thickness of the polyamide nanofilm supported on the silicon wafer was measured using the profilometer (DEKTAK-XT, Bruker GmbH, Germany). In this process, the polyamide composite membrane was immersed in DMF to remove the excess PES and nonwoven fabric, yielding the substrate-free polyamide layer. The resulting polyamide layers were then transferred onto a silicon wafer for thickness measurement.

**PIP diffusion capability evaluation**

The PIP diffusion capability was evaluated by the amount of PIP that diffused across the nascent layer. First, the solid amine layer was prepared on the ultrafiltration substrate, and the interfacial polymerization reaction was initiated at the corresponding TMC concentration. The reaction was terminated after 10 s, and the resulting sample was immersed in ultrapure water overnight to remove the unreacted PIP solids, yielding the nascent polyamide layer. Subsequently, an H‑type diffusion cell was used to measure the PIP transmembrane diffusion capability of the nascent layer. Specifically, the PIP feed solution concentration was 1 wt%, and the permeate was collected after thorough stirring for 30 min. An aliquot of the collected solution was mixed with alizarin red indicator, and the absorbance at 520 nm was measured using a UV‑vis spectrophotometer to quantitatively characterize the transmembrane diffusion performance of the nascent layer toward PIP.

**Nanofiltration performance evaluation**

The molecular weight cut-off (MWCO) was analyzed by filtrating the aqueous solutions of PEG with a series of molecular weights (200, 400, 600, 1000, 1500). The concentration of PEG is 0.10 wt%. The polyamide composite membrane was then evaluated by a laboratory-scale dead-end filtration module with an effective filtration area of 7.07 cm^2^. The solute retention (*R*, %) was calculated according to the following formula:

$R=\left( 1-\frac{C_{p}}{C_{f}} \right)\times100\%$	(S1)

where, *C_p_* (wt %) and *C_f_* (wt %) are the concentrations of filtrate solution and feed solution, respectively. The PEG concentration was measured by a total organic carbon analyzer (TOC, GE Sievers InnovOx ES, USA). Retention-molecular weight curves were obtained and then the MWCO of the composite membrane was identified from the curve point corresponding to a 90% of retention.

The nanofiltration performance of the polyamide composite membranes were assessed using a cross-flow filtration apparatus with the 0.1 wt % salt solution as the feed solution. The composite membranes were pre-compacted for 0.5 h under a pressure of 0.6 MPa. Then, salt rejection and water flux were measured at 30 ℃ with a cross-flow velocity of 30 L h^-1^. The salt rejection (*R*, %) was calculated by the formula 9, where *C_p_* (wt %) and *C_f_* (wt %) represent the concentration of salt in the filtrate and feed solutions, respectively. The water flux (*F*, L m^-2^ h^-1^) was calculated by formula *F* = *V*/*AT*, where *V* (L) represents the volume of filtrate collected over a membrane area of *A* (m^2^) during a period of *T* (h). The permeation flux (*L_p_*, L m^-2^ h^-1^ bar^-1^) was calculated by the formula *L*_p_ = *F*/*P*, where *P* (bar) is the pressure applied during the measurement.

**Figure S1** Structural model of interfacial polymerization.

**Figure S2** Nucleation process of the saturated PIP solution in hexane.

**Figure S3** Constant temperature and humidity control system used in the experiment.

**Figure S4** (A) Schematic diagram of the PIP nucleation and deposition process, and (B-E) photographs showing the surface morphology of the solid amine layer prepared under different temperature and humidity conditions. Preparation conditions for B: 10% RH and 25 ℃; for C: 20% RH and 25 ℃; for D: 30% RH and 25 ℃; for E: 20% RH and 35 ℃.

**Table S1** Surface morphology of the solid amine layer prepared under different temperature and humidity conditions.

| **T (℃)**  **RH (%)** | **20** | **25** | **30** | **35** |
| --- | --- | --- | --- | --- |
| 10 | Crystallized | Crystallized | Crystallized | Bare |
| 20 | Fairly uniform | Uniform | Fairly uniform | Bare |
| 30 | Fairly uniform | Speckled | Speckled | Bare |

**Figure S5** Grain size distribution of PIP crystals formed by evaporation at 25 °C and 20% RH. Specifically, the solid amine layer was prepared using the same method as that used to fabricate the polyamide composite membrane on the ultrafiltration support. A saturated PIP solution in hexane (5 mL) was introduced onto the surface of the ultrafiltration membrane with an effective area of 38.5 cm^2^ and allowed to evaporate under controlled conditions for 2 min. The residual liquid on the membrane surface was then collected, and the size of the PIP crystals in the resulting solution was characterized by dynamic light scattering.

**Figure S6** Loading mass of the solid amine layer on the substrate under different evaporation times.

**Figure S7** Surface roughness of the solid amine layer on different substrates at different evaporation times.

**Figure S8** Schematic diagram of monitoring interfacial monomer behavior at the solid-liquid interface.

**Figure S9.** QCM-D testing system.

**Figure S10.** Dissolution curve of the solid amine layer.

**Figure S11.** Fitted PIP flux under different interfacial monomer concentrations. The diffusion process within the open module was modeled using the COMSOL Multiphysics with the dilute species transport interface. The entire computational domain was filled with liquid hexane at 25 ℃, with a diffusion coefficient of 3.39×10^−9^ m^2^ s^-1^. At the initial time, the PIP concentration within the computational domain was set to 0, and a fixed concentration boundary condition was applied at the bottom. The diffusion behavior of PIP was simulated over 20 s with a time step of 0.1 s, and the PIP diffusion flux in the z-direction was statistically analyzed.

**Figure S12** *f* and *D* response curves of polyamide separation layer growth under seven different functional group ratio conditions.

**Figure S13** *f* and *D* response curves of polyamide separation layer growth at Stage 1 under seven different functional group ratio conditions.

**Figure S14** The apparent relative incorporation of TMC under seven different reaction functional group ratios.

**Figure S15** Schematic model of the diffusion experiment.

**Figure S16** Thickness of the nascent layers prepared under different reaction functional group ratio conditions.

**Figure S17** *f* and *D* response curves of polyamide separation layer growth at Stage 3 under seven different functional group ratio conditions.

**Table S2** Surface group analysis and statistics of HABD-related structural parameters of polyamide composite membranes prepared under different functional group ratios.

| **NH:COCl** | **C (%)** | **O (%)** | **N (%)** | **N2 (%)** | **N1+N3 (%)** | **O1 (%)** | **HABD (mmol g^-1^)** | **HCD^*^ (mmol g^-1^)** | **HAD (mmol g^-1^)** |
| --- | --- | --- | --- | --- | --- | --- | --- | --- | --- |
| 2:1 | 68.5 | 18.9 | 12.6 | 9.9 | 2.7 | 9.0 | 7.19 | 3.28 | 2 |
| 1.5:1 | 69.2 | 18.7 | 12.1 | 9.8 | 2.4 | 8.9 | 7.29 | 3.31 | 1.75 |
| 1:1 | 68.5 | 19.2 | 12.3 | 10.5 | 1.8 | 8.7 | 7.61 | 3.15 | 1.3 |
| 1:1.2 | 68.4 | 19.9 | 11.7 | 10.1 | 1.6 | 9.8 | 7.39 | 3.53 | 1.18 |
| 1:1.5 | 68.4 | 20.2 | 11.4 | 9.8 | 1.6 | 10.4 | 7.19 | 3.79 | 1.18 |
| 1:2 | 68.6 | 20.2 | 11.2 | 9.8 | 1.4 | 10.4 | 7.26 | 3.65 | 0.94 |
| 1:3 | 68.8 | 20.2 | 11.0 | 9.6 | 1.4 | 10.6 | 7.28 | 3.96 | 1.07 |

**^*^** HCD originates from the hydrolysis of acyl chloride during post‑treatment.

**Figure S18** Surface energy of composite membranes prepared under different functional group ratios.

**Figure S19** SEM images of the composite membranes prepared under different functional group ratios.

**Figure S20.** (A) MWCO, (B) zeta potential, (C) rejection performance of different salts and (D) water permeance of the composite membranes prepared by traditional liquid-liquid interfacial polymerization.

**References**

1. Xue, Y.-R.; Liu, C.; Ma, Z.-Y.; Zhu, C.-Y.; Wu, J.; Liang, H.-Q.; Yang, H.-C.; Zhang, C.; Xu, Z.-K., “Harmonic amide bond density as a game-changer for deciphering the crosslinking puzzle of polyamide.” Nature Communications 2024, 15 (1), 1539. https://doi.org/10.1038/s41467-024-45918-4
